# First person – Lena Marie Westermann

**DOI:** 10.1242/dmm.047860

**Published:** 2020-11-18

## Abstract

First Person is a series of interviews with the first authors of a selection of papers published in Disease Models & Mechanisms, helping early-career researchers promote themselves alongside their papers. Lena Marie Westermann is first author on ‘Imbalanced cellular metabolism compromises cartilage homeostasis and joint function in a mouse model of mucolipidosis type III gamma’, published in DMM. Lena Marie is an MD student in the lab of Dr Sandra Pohl at University Medical Center Hamburg-Eppendorf, Hamburg, Germany, investigating bone and connective tissue phenotypes in lysosomal storage disorders.


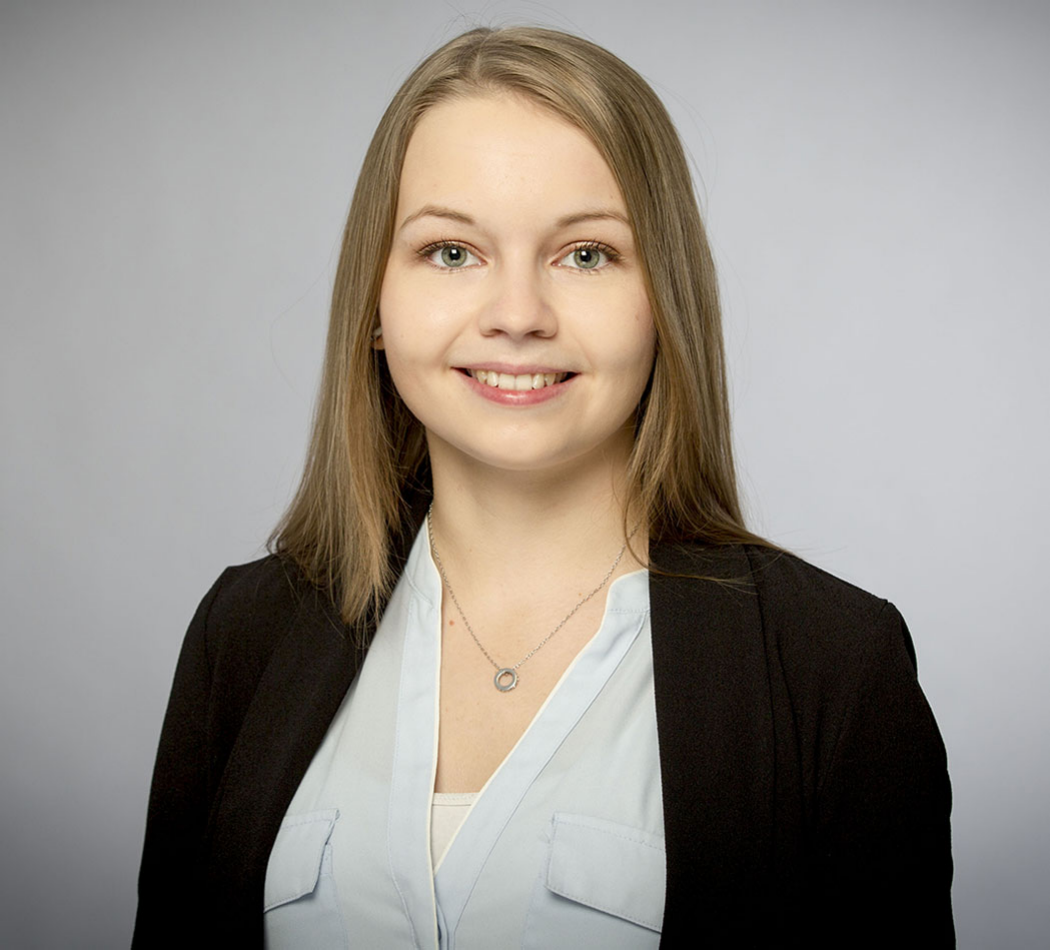


**Lena Marie Westermann**

**How would you explain the main findings of your paper to non-scientific family and friends?**

The lysosomal storage disorders mucolipidosis type II (MLII) and type III (MLIII) are rare genetic diseases affecting a part of the cell called the lysosome, working as a recycling facility in every body cell and degrading leftover cell material. Usually, the ‘facility workers’, the so-called lysosomal enzymes, are produced in another part of the cell and then transported to the lysosome, where they degrade the material. MLII and MLIII patients have a deficient enzyme, called phosphotransferase, which usually targets the lysosomal enzymes for this transport. For this reason, unnecessary cell material can no longer be efficiently degraded by lysosomes and eventually becomes toxic to the cell. Patients with MLII and MLIII are severely affected in almost every organ and suffer from enormous pain and limited joint mobility.

In our study, we found that joints in MLII and MLIII patients have differential mobility. So, we decided to investigate these phenomena in more detail using mouse models of the diseases, and performing various genetic and microscopic tissue analyses.

We figured out that, in the MLIII mouse model, cells of the cartilage do not develop and function normally. Moreover, we observed structural changes in the embedding tissue around cartilage cells. In addition to the cartilage, function of the tendon – another connective tissue component of the joint – also appeared to be impaired. Putting all these findings together, we got a better idea of the processes behind the clinical manifestations of these diseases, but further investigations will be necessary.

**What are the potential implications of these results for your field of research?**

Impairments of the locomotor system in lysosomal storage diseases are often described as disorders in bone homeostasis. This study suggests that joint cartilage and tendon tissue are also impaired in patients with MLII and MLIII. Thus, medical professionals should keep a better eye on patients’ cartilage and tissue, when it comes to disease monitoring and therapy. Moreover, the link between lysosomal deficiency and homeostasis of cartilage and extracellular matrix could be an interesting starting point for studies about other lysosomal storage disorders and even degenerative joint diseases in general. Nevertheless, further investigations are needed to identify more specific molecular pathomechanisms that could help us to develop new treatments.

**What has surprised you the most while conducting your research?**

During my scientific research about MLII and MLIII, I was mostly surprised at disparities in the pathology of these lysosomal storage diseases, even though both of them affect GlcNAc-1-phosphotransferase. That means that there have to be significant differences in the function of the subunits of this enzyme that we don't know of yet. I'm curious about future research on the function of the GlcNAc-1-phosphotransferase subunits and how we can use this knowledge to understand cell metabolism better and help patients with personalized treatment options.

“The most significant challenge in our research generation is the exponentially increasing amount of data.”

**Describe what you think is the most significant challenge impacting your research at this time and how will this be addressed over the next 10 years?**

The most significant challenge in our research generation is the exponentially increasing amount of data. Science becomes faster and we need to find a way to structure and exploit this knowledge. In my opinion, the best way is to build bigger research networks sharing their data in open resource databases and working together on a subject without rivalry. To realize that, the science community needs to rethink their quality criteria for successful research. Complementing the impact factor by some kind of cooperation index could be a start. Moreover, scientific journals should rework their publication guidelines to enable scientists to collaborate more, e.g. with multiple shared first and last authorships.

**Figure:**
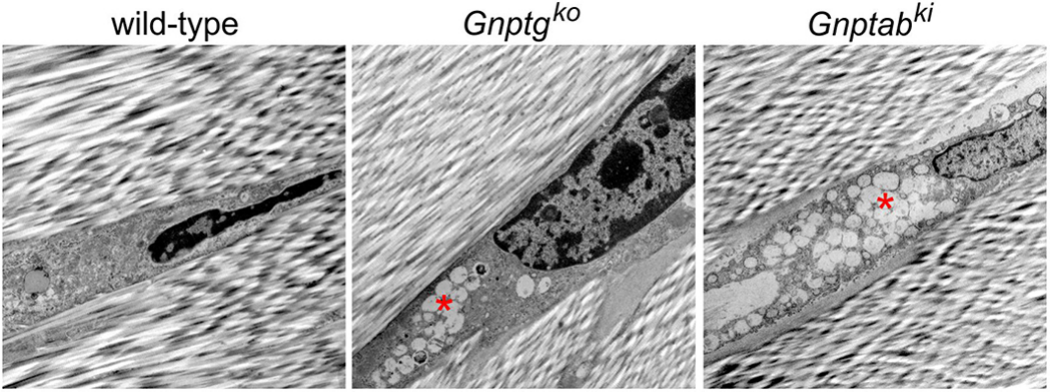
**Electron microscopy reveals enlarged lysosomes with non-degraded storage material in murine MLII and MLIII tenocytes.**

**What changes do you think could improve the professional lives of early-career scientists?**

I met a lot of, especially MD, students who gave up on their research even after a year of experimental wet lab work because they felt overchallenged with statistical analysis and writing their thesis. In my opinion, we need more structured doctoral programs accompanying the whole doctorate to improve scientific basic skills. Moreover, continuous supervision by the PI could additionally help to overcome insecurities, and mentoring programs with positive role models would complete that.

**What's next for you?**

After finishing my medical study at the end of this year and completing my MD thesis, I will start my residency in pediatrics. I would like to complement my current fundamental research about lysosomal storage disorders with further clinical and therapy-related studies.
